# Perceptions of chest pain and healthcare seeking behavior for chest pain in northern Tanzania: A community-based survey

**DOI:** 10.1371/journal.pone.0212139

**Published:** 2019-02-12

**Authors:** Julian T. Hertz, Deng B. Madut, Revogatus A. Tesha, Gwamaka William, Ryan A. Simmons, Sophie W. Galson, Francis M. Sakita, Venance P. Maro, Gerald S. Bloomfield, John A. Crump, Matthew P. Rubach

**Affiliations:** 1 Division of Emergency Medicine, Duke University Medical Center, Durham, North Carolina, United States of America; 2 Department of Medicine, Duke University Medical Center, Durham, North Carolina, United States of America; 3 Department of Statistical Science, Duke University, Durham, North Carolina, United States of America; 4 Kilimanjaro Christian Medical Centre, Moshi, Tanzania; 5 Duke Global Health Institute, Duke University, Durham, North Carolina, United States of America; 6 Department of Emergency Medicine, Kilimanjaro Christian Medical Centre, Moshi, Tanzania; 7 Department of Medicine, Kilimanjaro Christian Medical Centre, Moshi, Tanzania; 8 Division of Cardiology, Duke University Medical Center, Durham, North Carolina, United States of America; 9 Otago Global Health Institute, University of Otago, Dunedin, New Zealand; Medical University Graz, AUSTRIA

## Abstract

**Background:**

Little is known about community perceptions of chest pain and healthcare seeking behavior for chest pain in sub-Saharan Africa.

**Methods:**

A two-stage randomized population-based cluster survey with selection proportional to population size was performed in northern Tanzania. Self-identified household healthcare decision-makers from randomly selected households were asked to list all possible causes of chest pain in an adult and asked where they would go if an adult household member had chest pain.

**Results:**

Of 718 respondents, 485 (67.5%) were females. The most commonly cited causes of chest pain were weather and exercise, identified by 342 (47.6%) and 318 (44.3%) respondents. Two (0.3%) respondents identified ‘heart attack’ as a possible cause of chest pain. A hospital was selected as the preferred healthcare facility for an adult with chest pain by 277 (38.6%) respondents. Females were less likely to prefer a hospital than males (OR 0.65, 95% CI 0.47–0.90, *p* = 0.008).

**Conclusions:**

There is little community awareness of cardiac causes of chest pain in northern Tanzania, and most adults reported that they would not present to a hospital for this symptom. There is an urgent need for educational interventions to address this knowledge deficit and guide appropriate care-seeking behavior.

## Introduction

The symptom of chest pain is associated with serious conditions and is present in the large majority of patients with acute coronary syndrome (ACS) in high-income countries worldwide [1, 2]. Much less is known about the symptomatology of ACS in sub-Saharan Africa, but preliminary data from small single-center studies in the region have found that up to 84% of patients diagnosed with ACS also presented with chest pain [3, 4].

In recent years, sub-Saharan Africa has faced a dramatic rise in cardiovascular risk factors such as hypertension, diabetes, and obesity [5, 6]. In northern Tanzania, for example, the local prevalence of hypertension among adults has risen from 7% in 1993 [7] to 28% in 2014 [8]. Despite the well-documented surge in these risk factors, very little is known about the prevalence of ischemic heart disease across sub-Saharan Africa, and ACS remains a rare diagnosis among hospitalised adults [9]. In Tanzania, for example, ischemic heart disease is estimated to be the fourth leading cause of death based on extrapolation from multiple data sources [10], but there are no published reports empirically demonstrating the burden of ischemic heart disease in the country. If the projections for Tanzania are correct, there are many possible reasons for possible under-reporting of ACS in the region, including physician practices, local sociomedical culture, resource limitations, research priorities, systems challenges, and patient beliefs and behaviors.

To our knowledge, there have been no published studies regarding community perceptions of chest pain and healthcare seeking behavior for chest pain in sub-Saharan Africa. Nonetheless, understanding patient perceptions of ACS symptoms like chest pain and their patterns of care-seeking are an essential step in identifying barriers to ACS diagnosis and care in the region. If patients do not recognise symptoms of ACS as a reason to report to a hospital, then the burden of disease of ACS may be under-appreciated. Previous research regarding febrile illness in sub-Saharan Africa has demonstrated that patients often attributed fevers to non-biomedical causes such as weather changes, resulting in patients seeking care outside of the formal healthcare system, which likely results in underreporting of certain infectious diseases [11]. It is unknown whether or not similar patient beliefs and care-seeking behaviors are contributing to underreporting of ACS. If so, such beliefs and behaviors may be reinforcing a misperception that ACS is a relatively uncommon and unimportant disease in the region [12].

The aim of this study was to describe healthcare seeking behavior for adults with chest pain and identify common community explanations for chest pain among residents of northern Tanzania. To do so, we conducted a large cross-sectional community survey of adults in the Kilimanjaro Region.

## Methods

### Ethics statement

This study received ethics approval from the Duke Health Institutional Review Board, the Kilimanjaro Christian Medical Centre Research Ethics Committee, and the Tanzania National Institutes for Medical Research Ethics Coordinating Committee. Written informed consent was obtained from all participants.

### Setting

This study was performed in the Kilimanjaro Region of northern Tanzania. The study area included the city of Moshi (population 184,289 [13]) and the two surrounding rural districts, Hai (population 210,530 [13]) and Moshi Rural (population 466,740 [13]). The study location was selected for its known high prevalence of cardiovascular risk factors. The estimated local prevalence of hypertension was 28% and the estimated local prevalence of glucose impairment was 22% in 2014 [8, 14]. The dominant local tribe is the Chagga tribe.

### Sampling design

A two-stage randomized population-based cluster survey was performed with selection proportional to population size, following World Health Organization recommendations for vaccination coverage cluster surveys [15]. Within the study region, sixty villages were randomly selected in a population-weighted fashion. Twelve random points within each village were selected using Quantum Geographic Information System (QGIS, v2.18.7) and their global positioning system (GPS) coordinates were recorded. Each GPS location was then visited by the study team using Garmin eTrex handheld devices (Garmin, Olathe, Kansas) and the household nearest to the selected point was approached for inclusion in the study. If no one was available to participate in the survey at the closest household, then the next nearest household was approached.

### Survey translation

Survey questions were translated into Swahili and back-translated into English to ensure content clarity and fidelity. Because ‘chest pain’ can be a nebulous term, we piloted several word choice options with local Tanzanians with both medical and non-medical backgrounds, and we arrived at ‘maumivu ya kifua.’ Questions were independently back-translated in order to confirm fidelity to the essence of the question and to flag any potential ambiguity.

### Survey procedures

The study was conducted from February through May of 2018. Only individuals who self-identified as healthcare decision makers for the household were eligible for inclusion in the study. Respondents were asked in an open-ended fashion to list as many causes of chest pain in an adult that they could think of. They were not given options to choose from. They were then asked where they would present for care if they or another adult in their household were to have chest pain, from a list including common types of healthcare facilities in Tanzania, traditional healers, self-treatment at home, and watchful waiting. Sociodemographic information including age of respondent, household access to health insurance, and level of education of head of household was also collected. Surveys were administered in Swahili, and all responses were recorded using Open Data Kit software (ODK v1.12.2, Seattle, Washington) on Samsung Galaxy Tab A tablets (Samsung, Seoul, Korea). The final version of the survey instrument is provided in S1 File.

### Statistical analyses

Continuous variables are presented as means and standard deviations or medians and ranges, and categorical variables are presented as proportions. A socioeconomic status score was constructed using principal component analysis [16] from nine binary indicator variables: post-primary education, presence of electricity in the home, health insurance coverage, home floor material, ownership of a bank account, ownership of a car, ownership of a television, ownership of a refrigerator, and presence of a flush toilet in the home. Associations between categorical variables were analyzed with Pearson’s chi-squared, associations between categorical variables and continuous variables were analyzed with the t-test. Odds ratios and corresponding confidence intervals were calculated from contingency tables. Urban residence was defined as residence within Moshi Urban district. ‘Other heart problem’ was defined as any problem involving the heart identified by respondents other than a heart attack. The t-test was performed using STATA (v15.1, StataCorp, College Station, Tx); all other statistical analyses were performed using the R suite (v3.3.2, RStudio, Boston, MA).

## Results

A total of 718 respondents participated in the survey, with median (range) age of 48 (17–99) years. Table 1 presents the full demographic profile of participants. The majority of respondents were female (485, 67.5%), had primary school education (497, 69.2%), and did not have health insurance (488, 68.0%).

**Table 1:** Sociodemographic features of household survey respondents, Moshi Urban, Moshi Rural, and Hai Districts, 2018 (N = 718).

	n	(%)
Female	485	(67.5)
Urban residence	155	(21.6)
Education		
None	40	(5.6)
Primary	497	(69.2)
Secondary	132	(18.4)
Post-Secondary	49	(6.8)
Have health insurance	230	(32.0)
Religion		
Christian	584	(81.3)
Muslim	115	(16.0)
Other	19	(2.6)
Chagga tribe	535	(74.5)
	Median	(Range)
Age, years	48	(17, 99)
Household size, number of persons	4	(1, 13)
SES score	0.29	(0, 1.01)

SES: socioeconomic status

Table 2 presents the possible causes of chest pain in an adult identified by the participants. Weather and exercise were the most commonly mentioned causes of chest pain, cited by 342 (47.6%) and 318 (44.3%) respondents, respectively. Ninety-four (13.1%) participants were unable to think of any causes of chest pain. Two (0.3%) respondents identified ‘heart attacks’ and 5 (0.7%) respondents identified ‘other heart problems’ as possible causes of chest pain, respectively.

**Table 2:** Possible causes of chest pain in an adult identified by adult residents of northern Tanzania, 2018 (N = 718).

Cause	Number of respondents (%)
Weather	342 (47.6)
Exercise	318 (44.3)
Cigarette smoking	95 (13.2)
Dust	66 (9.2)
Tuberculosis	62 (8.6)
Food	53 (7.4)
Pneumonia	50 (7.0)
Other lung problems	42 (5.8)
Other infections	41 (5.7)
Smoke Inhalation	34 (4.7)
Alcohol	31 (4.3)
Injury	24 (3.3)
Allergy	8 (1.1)
Smells	5 (0.7)
Malaria	5 (0.7)
Other heart problems	5 (0.7)
High blood pressure	3 (0.4)
Heart attack	2 (0.3)
Others	36 (5.0)
Don’t know any	94 (13.1)

Table 3 presents the responses to the question, ‘Where would you seek care if you or another adult in your household had chest pain?’ The most commonly selected facility was a hospital, but the majority of respondents (441, 61.4%) said they would present somewhere other than a hospital. Only 104 (14.5%) participants said they would seek care entirely outside of the formal healthcare system, either by going directly to a pharmacy for treatment, self-treating at home, or watchful waiting. No respondent said they would go to a traditional healer.

**Table 3:** Responses to the question ‘Where would you seek care if you or another adult in your household had chest pain?’ among adults in northern Tanzania, 2018 (N = 718).

Facility	Number of respondents	(%)
Hospital	277	(38.6)
Dispensary	206	(28.7)
Health center	124	(17.3)
Pharmacy	60	(8.4)
Self-treatment at home	35	(4.9)
Do nothing/watchful waiting	9	(1.3)
Clinic	3	(0.4)
Traditional healer	0	(0.0)
Don’t know	4	(0.6)

Table 4 compares the sociodemographic characteristics of those who stated they would seek care at a hospital for chest pain versus those who did not. Females were significantly less likely than males to prefer seeking care at a hospital (OR 0.65, p = 0.008). There were otherwise no statistically significant associations observed between preference for a hospital and urban residence, education, ownership of health insurance, or age. Of the 6 respondents who identified either heart attacks or heart problems as possible causes of chest pain, one (16.7%) stated that they would present to a hospital for chest pain.

**Table 4:** Characteristics of respondents who would present to hospital for chest pain versus those who would not, northern Tanzania, 2018.

	Hospital first choice for chest pain n(%) (N = 277)	Hospital not first choice for chest pain n(%) (N = 441)	OR (95% CI)	*p*
Female	171 (61.7%)	314 (71.2%)	0.65 (0.47, 0.90)	0.008*
Urban residence	50 (18.1%)	105 (23.8%)	0.70 (0.48, 1.03)	0.068
Post-primary education	76 (27.4%)	105 (23.8%)	1.21 (0.86, 1.71)	0.276
Have health insurance	97 (35.0%)	133 (30.2%)	1.25 (0.91, 1.72)	0.174
Christian	229 (82.7%)	355 (80.5%)	1.16 (0.78, 1.71)	0.467
Chagga tribe	217 (78.3%)	318 (72.1%)	1.40 (0.98, 1.99)	0.062
Cited heart problem as possible cause of chest pain	1 (0.4%)	5 (1.1%)	0.32 (0.04, 2.72)	0.268
	Hospital first choice for chest pain mean(sd) (N = 277)	Hospital not first choice for chest pain mean(sd) (N = 441)		*p*
Age, years	49.7 (17.1)	47.0 (18.6)		0.054
SES score	0.37 (0.31)	0.34 (0.28)		0.245

SES: socioeconomic status

* *p* < 0.05

## Discussion

To our knowledge, this paper presents the first study of community perceptions of chest pain and healthcare seeking behavior for chest pain in sub-Saharan Africa. Only a tiny fraction of participants in this survey cited cardiovascular conditions as possible causes of chest pain, and the majority of respondents said they would not present to a hospital if they or another adult in their household had chest pain. If ischemic heart disease is as common in Tanzania as is currently estimated by the Global Burden of Disease study [10], then these findings highlight an urgent need for community education that is likely not unique to northern Tanzania.

Community awareness that life-threatening cardiovascular conditions like ACS could cause chest pain was extremely low in this study population. This finding stands in contrast to the results of multiple studies from a wide range of settings outside of Africa which found that large majorities of respondents recognised chest pain as potentially having a cardiac origin without being prompted by a picklist [17–19]. Thus, the findings of this study suggest that knowledge of ACS symptoms is much lower in northern Tanzania than in other settings across the globe. There has been no study of perceptions of chest pain elsewhere in sub-Saharan Africa, and additional research is needed to establish whether knowledge of ACS is similarly poor in other African communities. Increasing knowledge of ACS symptoms is an important public health goal because prior research has shown that such knowledge is associated with faster presentation to an appropriate healthcare facility [20].

A large number of participants in this study ascribed chest pain to environmental causes like weather, dust, and smoke inhalation. This finding is consistent with the results of other studies in sub-Saharan Africa that have described widespread community beliefs in weather conditions as a cause of other physical symptoms like fever [11, 21]. Infectious causes of chest pain, such as pneumonia, tuberculosis, and malaria, were also cited much more frequently by participants than cardiac causes. This difference may be reflective of the long-standing emphasis on infectious disease in this community, in terms of research, resources, and education. There are, however, no existing data about common causes of chest pain in Tanzania and data regarding the prevalence of ischemic heart disease in the country is sorely lacking. Thus, further research is needed to describe the actual causes of chest pain in Tanzania and local burden of ACS in order to determine the magnitude of the discrepancies between actual and perceived causes of chest pain.

Less than half of respondents reported that they would present to the hospital for chest pain, a preference that was prevalent across socioeconomic strata, tribal and religious affiliations, education levels, and urban and rural settings. This again stands in contrast to studies from outside Africa which have found that the majority of respondents would call an ambulance or present directly to the emergency department for chest pain [22]. Many participants in this study said they would seek care in other healthcare facilities such as dispensaries or health centers, but in the northern Tanzanian context such facilities would not be appropriate for ACS symptoms because they lack capacity for basic diagnostic testing such as electrocardiogram or cardiac biomarker testing. Women were less likely than men to state that they would present to a hospital. Such gender differences have been observed in some settings like Peru [17], but not in other settings like the United Kingdom [19]. Age was not a significant predictor of healthcare seeking behavior for chest pain in this study population, perhaps because many of the commonly cited explanations for chest pain such as weather and dust are not associated with age. Thus, there is a tremendous need for community educational interventions regarding appropriate care-seeking for chest pain in northern Tanzania, with particular attention to females, older residents, and other high-risk sub-populations. Such interventions would be more effective if they were supported by local burden of ischemic heart disease data, which are currently lacking. There have been no other studies of healthcare seeking behavior for chest pain in sub-Saharan Africa, and additional research is needed to establish whether similar patterns of care-seeking exist in other African settings.

This study had several limitations. First, participants were asked to report their care seeking behavior for a hypothetical case of chest pain rather than to report actual healthcare utilization during any prior episodes of chest pain. If respondents selected a hospital because they perceived it to be the most socially acceptable answer, this may have resulted in an overestimation in the true proportion of patients who would present to a hospital. Furthermore, participants were not given any specific options when asked to list causes of chest pain. This was done in an attempt not to bias responses to any set of ‘correct’ answers, but it is possible that some participants would have identified cardiac causes had they been present on a list of options. This may, therefore, have resulted in an underestimation of the proportion of patients who were aware of cardiac causes of chest pain. Additionally, patients were only asked to identify causes of chest pain generally, without specifying acuity or associated symptoms. Adding such details may have resulted in a larger proportion of respondents identifying cardiovascular causes of chest pain. Similarly, specifying acuity and associated symptoms may also have resulted in a larger proportion of respondents selecting a hospital as their preferred facility for chest pain. Finally, this survey was only given to self-identified healthcare decision makers. This was done in an attempt to survey only those whose opinions might guide actual healthcare seeking behavior, but exclusion of other adults may have resulted in a sample that was not truly representative of the local community.

### Conclusions

In conclusion, in northern Tanzania there was little community awareness that chest pain could be caused by cardiac pathologies, and the majority of respondents would not present to a hospital for chest pain. There is an urgent need for educational interventions to address this knowledge deficit and guide appropriate care seeking. As this was the first such study in sub-Saharan Africa, additional research is needed to describe perceptions of chest pain and healthcare seeking behavior for chest pain across the region.

## Supporting information

S1 File Perceptions of chest pain and healthcare seeking behavior questionnaire. (DOCX)

S1 Dataset Study data. (XLSX)
