# Supplementary material for: Perceptions of chest pain and healthcare seeking behavior for chest pain in northern Tanzania: A community-based survey
Source: PLoS One. 2019 Feb 12;14(2):e0212139. doi: 10.1371/journal.pone.0212139 (PMC6372176; doi:10.1371/journal.pone.0212139)
Supplement: S1 File — (DOCX) [file pone.0212139.s001.docx]

Perceptions of Chest Pain and Healthcare Seeking Behavior Questionnaire

Healthcare Utilization Survey

Northern Tanzania, 2018

[Swahili translation in brackets]

1. Please enter the surveyor's initials [Tafadhali weka herufi za mwanzo za majina ya mtafiti]:

2. Enter household ID [Ingiza namba ya kaya]:

3. District name [Jina la wilaya]:

4. Ward name [Jina la Kata]:

5. Village name [Jina la Kijiji]:

6. Are you the adult household member and decision-maker with respect to daily healthcare/healthcare utilization for the entire household and its members [Je wewe ni mtu mzima mwenye maamuzi katika familia hii katika masuala yanayohusu matumizi ya huduma za afya za kila siku kwa familia nzima]?
 ⎕ Yes [Ndio]
 ⎕ No [Hapana]

7. Provide the full name of the respondent [Andika majina kamili ya anayehojiwa]:

8. Provide the age (in YEARS) of the respondent [Umri wa anayehojiwa]:

9. Record the gender of the respondent [Jiinsia ya anaehojiwa].
 ⎕ Male [Kiume]
 ⎕ Female [Kike]

10. How many household members are living in this house in total (at the day of the visit) [Kuna jumla ya wakazi wangapi wanaoishi katika kaya hii]?

11. What are the causes of chest pain in an adult (Select Multiple) [Vitu gani vinaweza kusababisha maumivu ya kifua kwa watu wazima (chagua yote yaliyosahihi; jibu bila kuona majibu yanayofuata)]?
*Instructions: List all. Surveyor is not to offer suggestions or show the screen to the respondent.*
12. Record the FIRST CHOICE healthcare facility where you or an adult household member would seek care if they had chest pain [Andika chaguo la kwanza la kituo cha huduma za afya ambapo mkazi wa kaya hii angekwenda kutafuta huduma endapo angepata maumivu ya kifua].
*Instructions: Select one [Maelekezo: Chagua moja]* ⎕ Clinic [Kliniki]
 ⎕ Dispensary [Zahanati]
 ⎕ Health center [Kituo cha afya]
 ⎕ Hospital [Hospitali]
 ⎕ Pharmacy [Duka la dawa]
 ⎕ Self treatment [Kumtibu au kujitibu nyumbani]
 ⎕ Traditional healer [Mganga wa kienyeji]
 ⎕ Watchful waiting [Kutofanya chochote, kumwangalia au kujiangalia nyumbani]
 ⎕ Don’t know [Sijui]

13. If hospital selected, please specify the hospital [Tafadhali weka bayana hospitali].
 ⎕ Huruma Hospital
 ⎕ Kibosho Hospital
 ⎕ Kilema Hospital
 ⎕ KCMC
 ⎕ Marangu Hospital
 ⎕ Mawenzi Regional Hospital
 ⎕ Moshi Arusha Hospital
 ⎕ St. Joseph's Hospital
 ⎕ TPC Hospital
 ⎕ Other, specify: _______ [Nyingine, taja: ______]

14. Record the SECOND CHOICE healthcare facility where you or an adult household member would seek care if they had chest pain [Andika chaguo la pili la kituo cha huduma za afya ambapo mkazi wa kaya hii angekwenda kutafuta huduma endapo angepata maumivu ya kifua].
*Instructions: Select one [Maelekezo: Chagua moja]* ⎕ Clinic [Kliniki]
 ⎕ Dispensary [Zahanati]
 ⎕ Health center [Kituo cha afya]
 ⎕ Hospital [Hospitali]
 ⎕ Pharmacy [Duka la dawa]
 ⎕ Self treatment [Kumtibu au kujitibu nyumbani]
 ⎕ Traditional healer [Mganga wa kienyeji]
 ⎕ Watchful waiting [Kutofanya chochote, kumwangalia au kujiangalia nyumbani]
 ⎕ Don’t know [Sijui]

15. If hospital selected, please specify the hospital [Tafadhali weka bayana hospitali].
 ⎕ Huruma Hospital
 ⎕ Kibosho Hospital
 ⎕ Kilema Hospital
 ⎕ KCMC
 ⎕ Marangu Hospital
 ⎕ Mawenzi Regional Hospital
 ⎕ Moshi Arusha Hospital
 ⎕ St. Joseph's Hospital
 ⎕ TPC Hospital
 ⎕ Other, specify: _______ [Nyingine, taja: ______]

16. Where would you go if you or another adult in your home had shortness of breath [Ungeenda wapi kama wewe au mtu mzima mwingine angejisikia kupumua kwa shida]?
*Instructions: Select one [Maelekezo: Chagua moja]* ⎕ Clinic [Kliniki]
 ⎕ Dispensary [Zahanati]
 ⎕ Health center [Kituo cha afya]
 ⎕ Hospital [Hospitali]
 ⎕ Pharmacy [Duka la dawa]
 ⎕ Self treatment [Kumtibu au kujitibu nyumbani]
 ⎕ Traditional healer [Mganga wa kienyeji]
 ⎕ Watchful waiting [Kutofanya chochote, kumwangalia au kujiangalia nyumbani]
 ⎕ Don’t know [Sijui]

17. If hospital selected, please specify the hospital [Tafadhali weka bayana hospitali].
 ⎕ Huruma Hospital
 ⎕ Kibosho Hospital
 ⎕ Kilema Hospital
 ⎕ KCMC
 ⎕ Marangu Hospital
 ⎕ Mawenzi Regional Hospital
 ⎕ Moshi Arusha Hospital
 ⎕ St. Joseph's Hospital
 ⎕ TPC Hospital
 ⎕ Other, specify: _______ [Nyingine, taja: ______]

18. What is the highest completed level of formal school education of the head of the household? [Mkuu wa kaya ana elimu ya kiwango gani]?
 ⎕ No education
 ⎕ Some primary school
 ⎕ Completed primary school
 ⎕ Some secondary school
 ⎕ Completed secondary school
 ⎕ Some college
 ⎕ Completed college

19. What is the main occupation of the head of the household? [Mkuu wa kaya anafanya kazi gani]?
*Instructions: Select one [Maelekezo: Chagua moja]*
 ⎕ Driver [Dereva]
 ⎕ Guard/police [Polisi]
 ⎕ Healthcare worker [Mhudumu wa afya]
 ⎕ House girl [Msichana wa kazi]
 ⎕ Housewife [Mama wa nyumbani]
 ⎕ Livestock keeper [Mfugaji]
 ⎕ Unskilled laborer [Wafanyakazi wasio na ujuzi wa kusomea]
 ⎕ Street vendor [Machinga]
 ⎕ Religious occupation [Mhudumu wa dini]
 ⎕ Farmer [Mkulima]
 ⎕ Student [Mwanafunzi]
 ⎕ Teacher [Mwalimu]
 ⎕ Tradesperson [Fundi]
 ⎕ Unemployed [Hajaajiriwa]
 ⎕ Office worker [Mfanyakazi wa ofisini]
 ⎕ Small business manager [Mjasiriamali]
 ⎕ Business Owner [Mfanyabishara]
 ⎕ Other, specify: _______ [Nyingine, taja: ______]

20. What is the floor of your home made of [Je, sakafu ya nyumba hii imejengwa kwa kutumia malighafi gani]?
 ⎕ Brick [Tofali]
 ⎕ Cement/concrete [Simenti/zege]
 ⎕ Tiles [Vigae]
 ⎕ Mud [Tope]
 ⎕ Wood [Mbao]
 ⎕ Straw/leaves [Majan/matawi]
 ⎕ Metal/tin [Bati/debe]
 ⎕ Other, specify: _______ [Nyingine, taja: ______]
 ⎕ Don’t know [Sijui]

21. What is the wall of your home made of [Je, kuta za nyumba hii zimejengwa kwa kutumia malighafi gani]?
 ⎕ Brick [Tofali]
 ⎕ Cement/concrete [Simenti/zege]
 ⎕ Tiles [Vigae]
 ⎕ Mud [Tope]
 ⎕ Wood [Mbao]
 ⎕ Straw/leaves [Majan/matawi]
 ⎕ Metal/tin [Bati/debe]
 ⎕ Other, specify: _______ [Nyingine, taja: ______]
 ⎕ Don’t know [Sijui]

22. What is the roof of your home made of [Je, paa la nyumba hii limejengwa kwa kutumia malighafi gani]?
 ⎕ Brick [Tofali]
 ⎕ Cement/concrete [Simenti/zege]
 ⎕ Tiles [Vigae]
 ⎕ Mud [Tope]
 ⎕ Wood [Mbao]
 ⎕ Straw/leaves [Majan/matawi]
 ⎕ Metal/tin [Bati/debe]
 ⎕ Other, specify: _______ [Nyingine, taja: ______]
 ⎕ Don’t know [Sijui]

23. Do you have access to electricity in your household [Je, kuna umeme kwenye nyumbani hii]?
 ⎕ Yes [Ndio]
 ⎕ No [Hapana]
 ⎕ Don’t know [Sijui]

24. Which of the following items do members of this household own [Kipi kati ya vitu vifuatavyo kinamilikiwa na wakazi wa kaya hii]?
*Instructions: Choose all that apply [Maelekezo: Chagua zote zinazohusika]* ⎕ Radio [Redio]
 ⎕ Television [Televisheni]
 ⎕ Mobile phone [Simu ya mkononi]
 ⎕ Iron [Pasi]
 ⎕ Refrigerator [Friji]
 ⎕ Motorcycle [Pikipiki]
 ⎕ Car or truck [Gari]
 ⎕ Bank account [Akounti/Akiba ya benki]
 ⎕ Tap water [Maji ya bomba]
 ⎕ None of the above [Hakuna kati ya vilivyotajwa]

25. Does anyone in this household have any kind of health insurance [Je,wewe ama mkazi yeyote anahudumiwa na bima ya afya]?
 ⎕ Yes [Ndio]
 ⎕ No [Hapana]
 ⎕ Don’t know [Sijui]

26. If yes, Which of the following health insurance plans are members of this household members enrolled in [Wanafamilia wana tumia aina gani ya bima ya afya]?
*Instructions: Choose all that apply [Maelekezo: Chagua zote zinazohusika]* ⎕ National Health Insurance Fund (NHIF) [Mfuko wa taifa wa Bima ya Afya]
 ⎕ Social Health Insurance Benefits (SHIB) [Mfuko wa Bima ya afya ya jamii]
 ⎕ Community Health Fund (CHF) or Tiba Kwa Kadi (TIKA)
 ⎕ Private insurance [Bima ya binafasi]
 ⎕ Other, specify: _______ [Nyingine, taja: ______]
 ⎕ Don’t know [Sijui]

27. Which religion(s) do members of this household belong to [Je, wakazi wa kaya hii wana amini katika dini ipi]?
 ⎕ Christian [Ukristo]
 ⎕ Muslim [Uislamu]
 ⎕ Hindu [Hindu]
 ⎕ None [Hakuna]
 ⎕ Other, specify: _______ [Nyingine, taja: ______]

28. What tribes do members of this household belong to [Je,wakazi wa kaya hii ni kabila gani]?
 ⎕ Chagga
 ⎕ Pare
 ⎕ Sambaa
 ⎕ Masaai
 ⎕ Meru
 ⎕ Other, specify: _______ [Nyingine, taja: ______]

29. What type of toilet do members of this household usually use [Kwa kawaida ni aina gani ya choo ambacho hutumiwa na wakazi wa kaya hii]?
 ⎕ Flush toilet [Choo cha maji (ndani ya nyumba)]
 ⎕ Pit latrine [Choo cha shimo]
 ⎕ Free range [Popote nje]
 ⎕ Other, specify: _______ [Nyingine, taja: ______]
